# Experiences of public pool lifeguards: a qualitative study

**Published:** 2022-01

**Authors:** Parand Pourghane, Sanaz Salimi

**Affiliations:** ^*a*^ Associate Professor, Social Determinants of Health Research Center, Guilan University of Medical Sciences, Rasht, Iran; Associate Professor, Department of Nursing, Zeynab (P.B.U.H) School of Nursing and Midwifery, Guilan University of Medical Sciences, Rasht, Iran.; ^*b*^ Resident of Clinical Pharmacy, Mazandaran University of Medical Sciences, Sari, Iran.

## Abstract

**Background:**

Public swimming pools have received a lot of attention in recent years. Spatial standards and the presence of lifeguards with knowledge of the cardiopulmonary resuscitation process can be a safe and attractive environment. Lifeguards in swimming pools play a very important role in this process as their role has changed a lot over the last 15 years. The purpose of this study is to explore the experiences of lifeguards in the pools of East of Guilan to understand their positive and negative experiences, to promote their positive experiences, and to reduce or eliminate barriers and challenges at work in order to create a suitable environment for leisure activities and sports for people.

**Methods:**

This research was conducted qualitatively using a content analysis approach. For data collection, semi-structured interviews were conducted on 23 lifeguards who were selected through purposive sampling. Data analysis was performed according to the steps of Graneheim and Lundman. The accuracy and robustness of the research were assessed based on Lincoln and Guba's criteria.

**Results:**

Three main categories including "declining motivation", "remote accesses" and "educational/learning challenges" were extracted.

**Conclusions:**

The participants’ declining motivation was due to not paying attention to the capabilities of individuals and equating the experiences of people with low and high working experience, receiving late and insignificant salaries, and insufficient attention to their overtime which should be considered by the authorities. Another unpleasant experience for some participants was the far distance between pools and medical centers and hospitals which is a high-stress challenge for lifeguards in emergency situations. The presence of experienced lifeguards with more skills in the field of cardiopulmonary resuscitation, and early responses from emergency centers in the city can not only reduce the stress of lifeguards but also be the ideal solution for the survival of the drowned.

**Keywords:**

Public Pool, Lifeguards, Qualitative Study

